# Supplementary material for: Epoxidation of Methyl Esters as Valuable Biomolecules: Monitoring of Reaction
Source: Molecules. 2023 Mar 21;28(6):2819. doi: 10.3390/molecules28062819 (PMC10053758; doi:10.3390/molecules28062819)
Supplement: Supplementary file 1 [file molecules-28-02819-s001.zip › molecules-2243631-SI.pdf]

Epoxidation of Methyl Esters as Valuable Biomolecules: Monitoring of Reaction

Martin Hájek [1], Tomáš Hájek [2,*], David Kocián [1], Karel Frolich [1], András Peller [3]

[1] Department of Physical Chemistry, Faculty of Chemical Technology, University of Pardubice, Studentská 95, 532 10 Pardubice, Czech Republic
[2] Department of Analytical Chemistry, Faculty of Chemical Technology, University of Pardubice, Studentská 95, 532 10 Pardubice, Czech Republic
[3] Faculty of Chemical and Food Technology, Slovak University of Technology in Bratislava, Radlinského 9, 81237 Bratislava, Slovakia
[*] Corresponding author: Tomas.Hajek@upce.cz

Figure S1: ^{1}H NMR for methyl esters and epoxidized methyl esters of *Camelia sativa* oil.

methyl esters

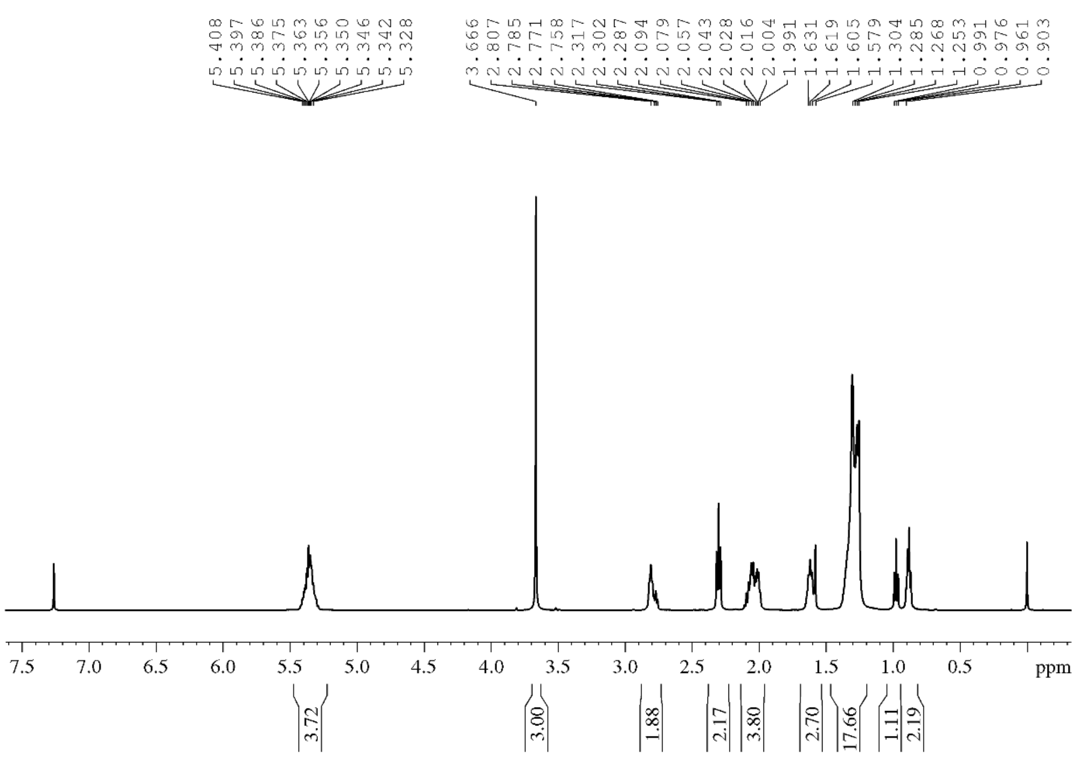

epoxidized methyl esters

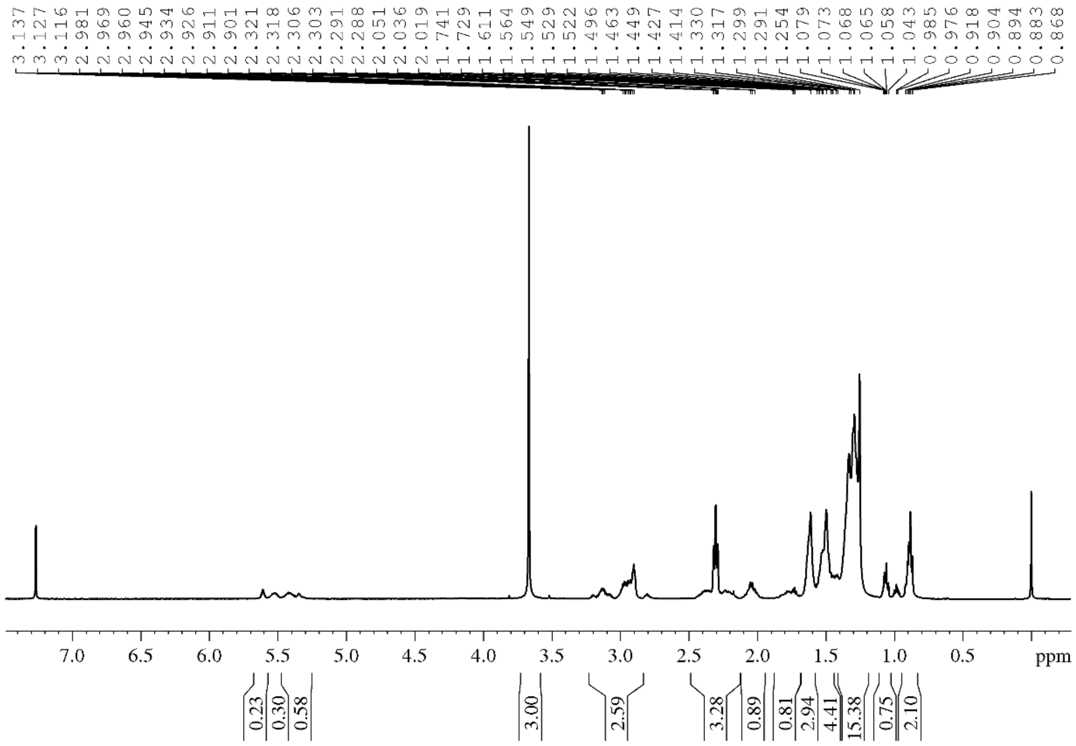

Table S1. Retention time (t_r) of fatty acid methyl esters and products of epoxidation

Compounds	t_r (min)
Methyl esters	
C16	16.95
C17 IS	18.31
C18:0	19.78
C18:1	20.45
C18:2	21.56
C18:3	22.97
C20:0	23.12
C20:1	23.91
C20:2	25.21
C22:0	26.91
C22:1	27.81
C24:0	31.30
products of epoxidation	
C18:1 1-Ep	32.17
C18:2 1-Ep I	33.08
C18:2 1-Ep II	33.26
C18:3 1-Ep I	34.60
C18:3 1-Ep II	35.11
C18:3 1-Ep III	35.27
C20:1 1-Ep	36.82
C20:2 1-Ep I	37.81
C20:2 1-Ep I	38.07
C22:1 1-Ep	42.24
C18:2 2-Ep I	48.85
C18:2 2-Ep II	49.26
C18:3 2-Ep I	52.19
C18:2 2-Ep III	53.71
C18:3 2-Ep II	56.58
C18:3 2-Ep III	57.14
C18:3 2-Ep IV	58.39
